# Differential Expression and Bioinformatics Analysis of circRNA in Non-small Cell Lung Cancer

**DOI:** 10.3389/fgene.2020.586814

**Published:** 2020-11-24

**Authors:** Qiuwen Sun, Xia Li, Muchen Xu, Li Zhang, Haiwei Zuo, Yong Xin, Longzhen Zhang, Ping Gong

**Affiliations:** ^1^School of Medical Imaging, Xuzhou Medical University, Xuzhou, China; ^2^Department of Radiation Oncology, Affiliated Hospital of Xuzhou Medical University, Xuzhou, China; ^3^School of Information and Control Engineering, University of Mining and Technology, Xuzhou, China; ^4^School of Medical Information and Engineering, Xuzhou Medical University, Xuzhou, China; ^5^Cancer Institute of Xuzhou Medical University, Xuzhou, China

**Keywords:** circRNA, expression, pathway, bioinformatics, non-small cell lung cancer

## Abstract

Circular RNA (CircRNA) plays an important role in tumorigenesis and progression of non-small cell lung cancer (NSCLC), but the pathogenesis of NSCLC caused by circRNA has not been fully elucidated. This study aimed to investigate differentially expressed circRNAs and identify the underlying pathogenesis hub genes of NSCLC by comprehensive bioinformatics analysis. Data of gene expression microarrays (GSE101586, GSE101684, and GSE112214) were downloaded from Gene Expression Omnibus (GEO) database. Differentially expressed circRNAs (DECs) were obtained by the “limma” package of R programs and the overlapping operation was implemented of DECs. CircBase database and Cancer-Specific CircRNA database (CSCD) were used to find miRNAs binding to DECs. Target genes of the found miRNAs were identified utilizing Perl programs based on miRDB, miRTarBase, and TargetScan databases. Functional and enrichment analyses of selected target genes were performing using the “cluster profiler” package. Protein-protein interaction (PPI) network was constructed by the Search Tool for the STRING database and module analysis of selected hub genes was performed by Cytoscape 3.7.1. Survival analysis of hub genes were performed by Gene Expression Profiling Interactive Analysis (GEPIA). Respectively, 1 DEC, 249 DECs, and 101 DECs were identified in GSE101586, GSE101684, and GSE112214. A total of eight overlapped circRNAs, 43 miRNAs and 427 target genes were identified. Gene Ontology (GO) enrichment analysis showed these target genes were enriched in biological processes of regulation of histone methylation, Ras protein signal transduction and covalent chromatin modification etc. Pathway enrichment analysis showed these target genes are mainly involved in AMPK signaling pathway, signaling pathways regulating pluripotency of stem cells and insulin signaling pathway etc. A PPI network was constructed based on 427 target genes of the 43 miRNAs. Ten hub genes were found, of which the expression of MYLIP, GAN, and CDC27 were significantly related to NSCLC patient prognosis. Our study provide a deeper understanding the circRNAs-miRNAs-target genes by bioinformatics analysis, which may provide novel insights for unraveling pathogenesis of NSCLC. MYLIP, GAN, and CDC27 genes might serve as novel biomarker for precise treatment and prognosis of NSCLC in the future.

## 1. Introduction

Lung cancer has become one of the most serious malignant tumors in the world. The incidence rate of lung cancer ranks first among men and women in second place (Hu et al., 2018). There are two types of lung cancer, non-small cell lung cancer (NSCLC), and small cell carcinoma (SCLC). NSCLC accounts for approximately 85% and about 75% of NSCLC patients were in the advanced stage when they were discovered. Despite new developments for NSCLC in diagnosis and treatment, the overall survival rate remains poor and patients with advanced or metastatic have a worse prognosis (Cheung and Juan, 2017). Chemotherapy and biological targeted therapy are the best ways for the advanced stage patients of NSCLC. Thus, the identification of effective biomarkers or therapeutic targets of NSCLC is of great significance in reducing mortality and improving clinical prognosis (Hong et al., 2015).

Circular RNAs (circRNAs) are a special type of non-coding RNA molecule, which has brought great interests to researchers. Recently, circRNA has become a hotspot in RNA and transcriptome research. Compared with the traditional liner RNA, circRNA molecule has a closed circular structure and is not affected by RNA exonucleases (Chen et al., 2019). The expression of circRNAs is more stable and less prone to degradation. Studies have shown that circRNA is rich in microRNA (miRNA) binding sites and can act as miRNA sponges in cells, which can abolish the inhibition of miRNAs on their target genes and increase the expression levels of target genes (Hansen et al., 2013; Zhang et al., 2018). Hansen et al. previously identified a highly expressed circRNA in human and mouse brain. In their study, CIRS-7 acts as a miR-7 sponge and term this circRNA as circular transcript ciRS-7 (circRNA sponge for miR-7). CiRS-7 contains more than 70 selectively conserved miRNA target sites and it can strongly suppress the activity of miR-7, resulting in increased expression levels of miR-7 targets (Hansen et al., 2013). Emerging evidence suggest that circRNAs are closely related to human diseases, especially cancers, and can serve as better biomarkers because of their abundance and stability. For example, Yang et al. (2020). found that circPTK2 exert critical roles in colorectal cancer (CRC) growth and metastasis, it may serve as a potential therapeutic target for CRC metastasis. The same study also revealed that circPTK2 regulates OGD-activated microglia-induced neuronal apoptosis via miR-29b-SOCS-1-JAK2/STAT3-IL-1βsignaling (Wang H. et al., 2019). In lung cancer, a group of circRNAs have also been found to be significantly dysregulated and several NSCLC-related circRNAs are identified. For example, Cheng et al. revealed that circTP63 was upregulated in lung squamous cell carcinoma (LUSC) tissues and its upregulation was correlated with larger tumor size and higher TNM stage in LUSC patients. *In-vitro* and *in-vivo*, elevated circTP63 promotes cell proliferation. Mechanistically, circTP63 shares miRNA response elements with FOXM1 (Cheng et al., 2019). Similarly, circPTK2 was significantly downregulated in NSCLC, circPTK2 over expression augmented TIF1γexpression, inhibited TGF-β-induced EMT and NSCLC cell invasion, whereas circPTK2 knockdown had the opposite effects (Wang et al., 2018). With the development of bioinformatics technology, there are more and more bioinformatics researches based on circRNA. However, the novel circRNAs and the function they played were still needed to explore.

**Figure 1:**
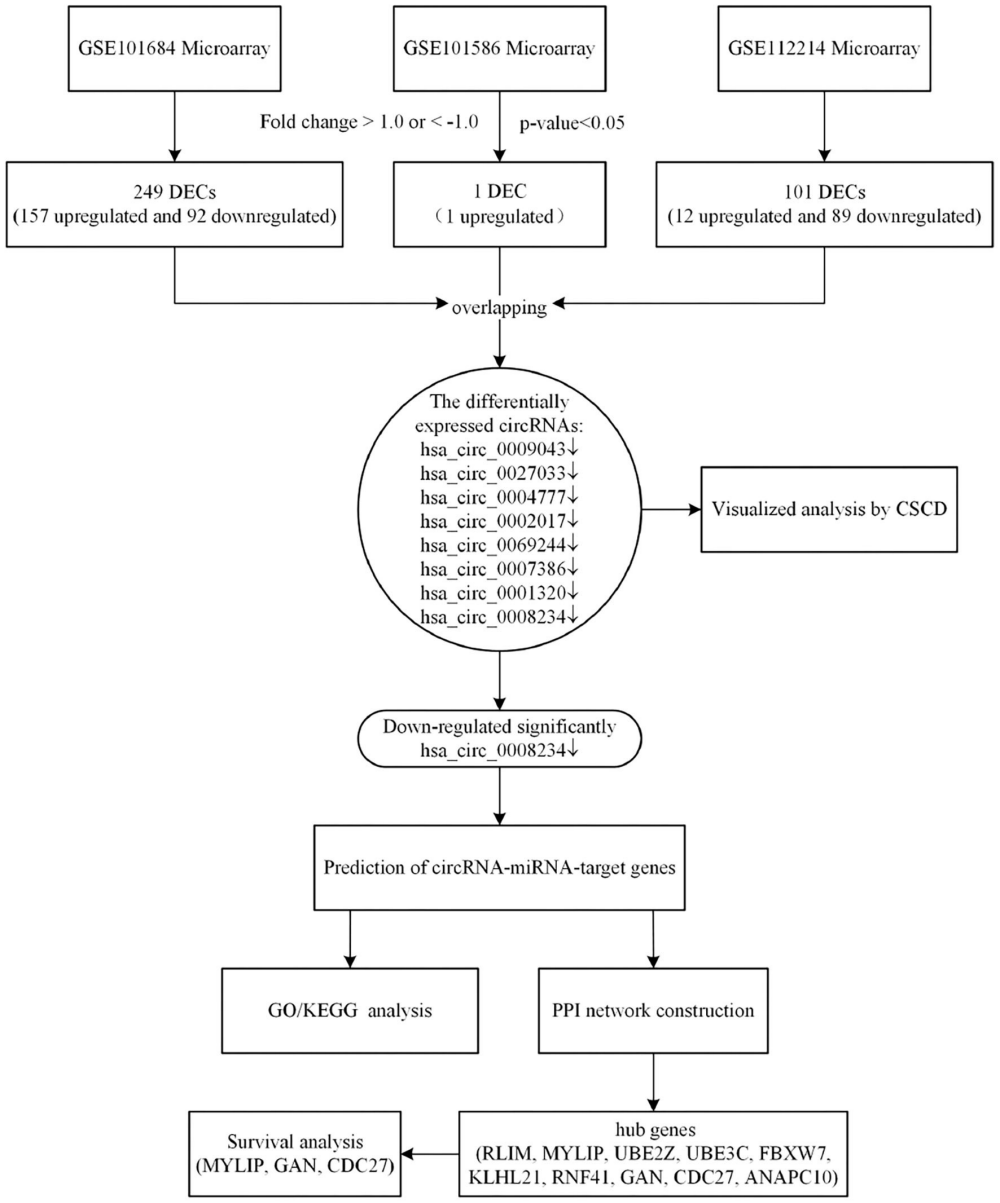
The bioinformatics workflow of this study.

In the present study, we analyzed the latest expression profile of circRNAs in lung cancer by using complex and comprehensive bioinformatics methods (Qu et al., 2018). According to the biological function of circRNA, the miRNA and target genes that bind to circRNA were predicted. Enrichment analysis of target genes provided novel insights in the treatment and prognosis of NSCLC. Besides, we performed the survival analysis to identify whether hub genes could be used as prognosis biomarkers for NSCLC. We collected the expression microarrays of circRNAs in NSCLC from Gene Expression Omnibus (GEO) datasets. Differentially expressed circRNAs (DECs) were identified by “limma” package in R software. Subsequently, the miRNAs binding to the significantly downregulated expressed circRNA were obtained by CSCD database. After predicting the miRNA-target genes, we evaluated the main functional pathways by Gene Ontology (GO) enrichment analyses and Kyoto Encyclopedia of Genes and Genomes (KEGG) pathway analyses. Based on the differentially expressed circRNAs-miRNAs-target genes identified, the protein-protein interaction (PPI) network was constructed by the Search Tool for the Tetrieval of Interacting Genes (STRING) database, then the visualization was performed by Cytoscape 3.7.1. Finally, Gene Expression Profiling Interactive Analysis (GEPIA) was used to analyse the survival of hub genes. These results provide a new direction for the mechanism and clinical treatment of NSCLC (Zhang et al., 2017). A flowchart of this study is shown in Figure 1.

## 2. Materials and Methods

### 2.1. Data Acquisition

The Gene Expression Omnibus database (GEO, https://www.ncbi.nlm.nih.gov/geo/) plays an important role in many fields, including comparative genomic analysis, proteomics, non-coding RNA, single nucleotide polymorphism genome and gene methylation status analysis (Liu et al., 2017; Sayers et al., 2018). A search for the microarray that met the requirements from the GEO dataset was performed by using “lung cancer” OR “lung tumor” AND “circRNA.” The inclusion and exclusion criteria were shown as follows: (1) microarray expression profiling of circRNAs, (2) including human NSCLC lung samples and matched adjacent samples, (3) raw matrix data and platform annotation data. After excluding the datasets with the latest time, 3 datasets [GSE101586 (GPL19978 platform), GSE101684 (GPL21825 platform), GSE112214 (GPL19978 platform)] were downloaded.

### 2.2. Data Processing

The raw microarray data were read by the R software and the expression values of the circRNAs were subjected to a base 2 logarithmic conversion. The same circRNAs in the expression matrix were combined by averaging the expression values. The circRNAs probe information were annotated by the platform file GPL21825 and GPL19978 (platform file of the microarrays). All related annotation such as probe name, platform information and sample information etc. were removed and the related gene expression level such as gene name, sample name were kept of the obtained original microarray data.

### 2.3. Identification of Differentially Expressed circRNAs

The ID of the gene matrix data were converted to gene names by Perl script. In order to minimize the heterogeneity among different datasets, normalization and log2 conversion were applied for the raw microarray data by using the “limma” package in the R software in our analyses (Liu et al., 2017). The same circRNAs in the expression matrix were combined by averaging the expression values. Subsequently, the fold-change and Student's t testing were used for screening for DECs in the three sets, respectively. The criterion for DEGs was |*log*_2_*FoldChange*| > 1 and the adjusted *P*−*value* < 0.05. Furthermore, volcano plots were performed based on the circRNAs information (Tian et al., 2020).

### 2.4. Prediction of circRNA-miRNA-Target Gene Interactions

The circBase database and Cancer-Specific CircRNA database (CSCD, https://gb.whu.edu.cn/CSCD/) were used to predict miRNA binding sites (MREs) (Rong et al., 2017), CircBase provided the chromosomes location of the circRNAs, the chromosomal location and length of the RNA required for the study. Interactions between miRNA and target genes were predicted based on the miRDB, miRTarBase, and TargetScan databases. Only target genes recognized by all three databases were considered as candidate target genes (Xiong et al., 2018).

### 2.5. GO and KEGG Pathway Enrichment Analyses

In order to understand the potential functions of circRNAs, GO, and KEGG pathway enrichment analysis of target genes were performed by using the “cluster profiler” package in the R software. GO analysis cover three areas, cellular components (CC), molecular functions (MF), and biological processes (BP), each category explains the biological function of genes at different levels (Zhang et al., 2019). The KEGG pathway enrichment analysis is used for the degree of enrichment of differential genes in pathway terms.

### 2.6. Construction PPI Network and Module Analysis

Based on the target genes identified, the PPI network was constructed by using the STRING database and the visualization was performed by Cytoscape 3.7.1 (Doncheva et al., 2019). The confidence score was set as *score* > 0.4. The Molecular Complex Detection (MCODE) and Cytohubba in Cytoscape were used to screen modules of hub genes from the PPI network with *MCODEscore* > 2 and *Cytohubbadegrees* > 10.

### 2.7. Survival Analysis of Hub Genes

GEPIA (http://gepia.cancer-pku.cn/) is an online tool for analyzing gene expression in cancer and healthy samples, which we used to analyze the following survival times: 50, 100, 150, 200, and 250 months (Tang et al., 2017). We performed the survival analysis to identify whether hub genes could be used as prognosis biomarkers for NSCLC.

## 3. Results

### 3.1. CircRNA Profile Overview

Data from each microarray of NSCLC and adjacent normal mucosa tissues were separately analyzed by R program to screen differentially expressed circRNAs. Overall, there were 249 DECs in GSE101684, of which expression levels of 157 circRNAs were increased (|*log*_2_*FoldChange*| > 1, *p* < 0.05) and 92 circRNAs were decreased (|*log*_2_*FoldChange*| > 1, *p* < 0.05). There was only 1 DEC in GSE101586 and it was up-regulated.

**Figure 2:**
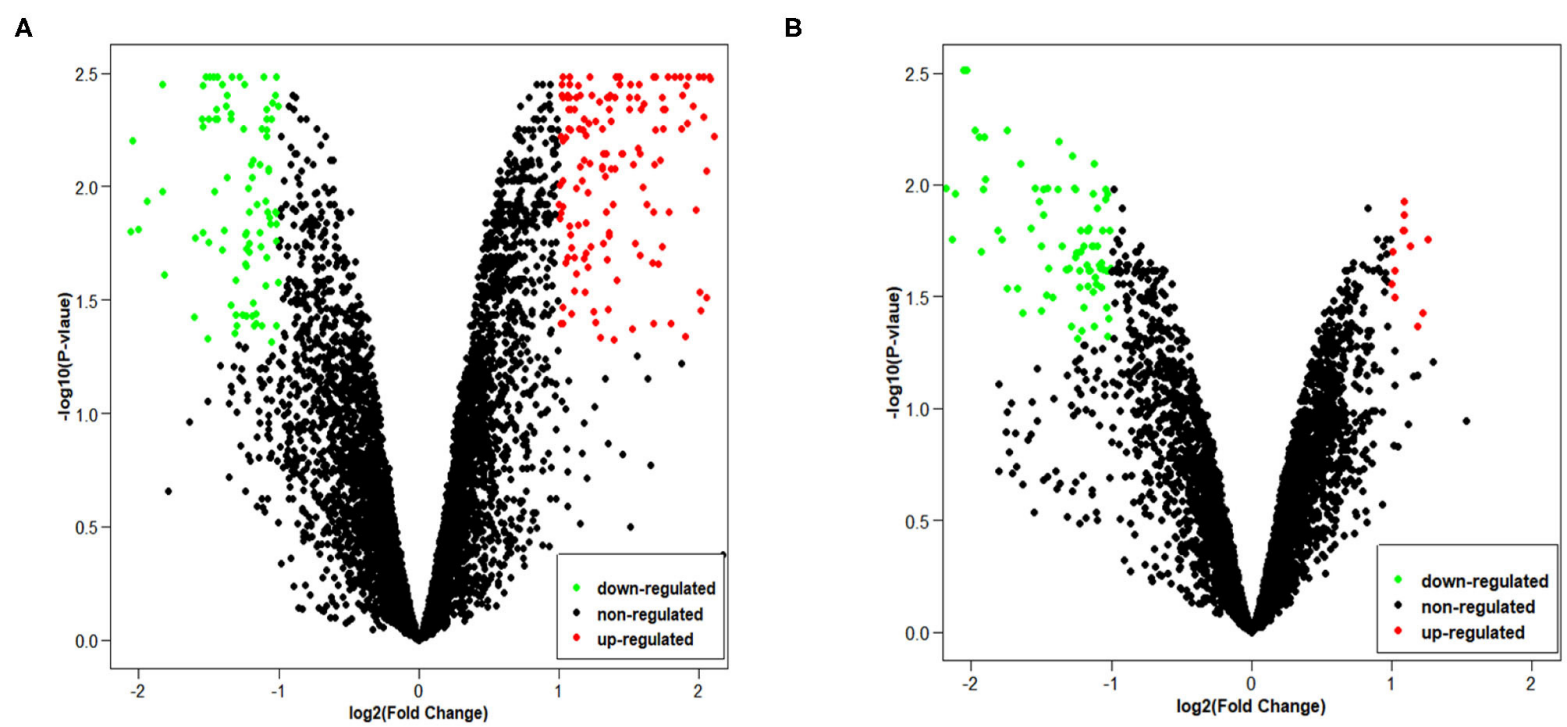
The volcano plots of two chips: **(A)** GSE101684, **(B)** GSE112214.

Since GSE101586 dataset has 1 DEC, we only provide volcano maps of GSE101684 and GSE112214 (Figure 2). The volcano map shows the number and distribution of the probes in the same plane, the horizontal axis represents the normalized difference (tumor group/normal group), the red dot indicates the up-regulated circRNAs and the green dot indicates the down-regulated circRNAs. DECs were classified and circRNA expression level were evaluated of different samples by pheatmap.

**Figure 3:**
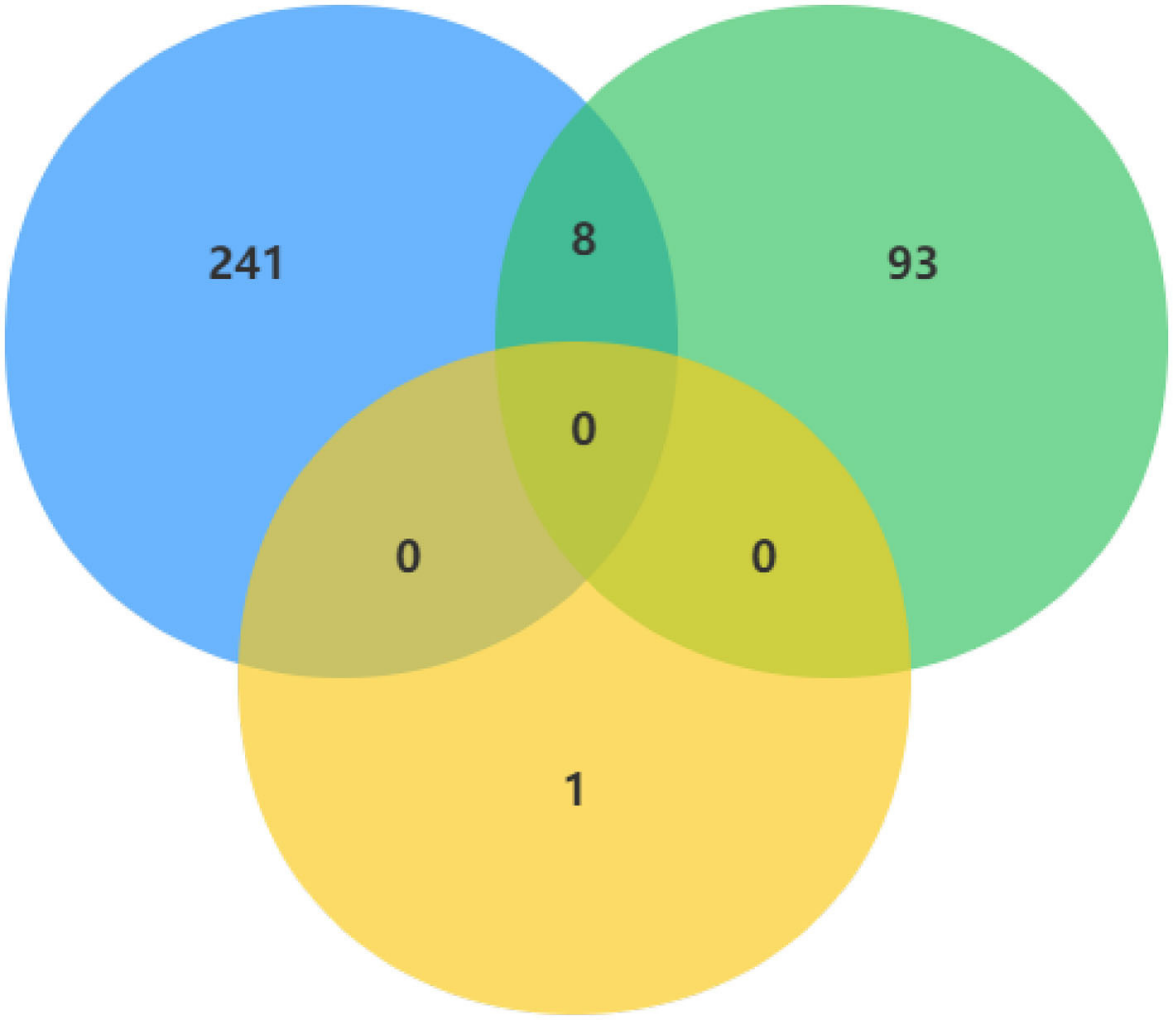
DECs were obtained by overlapping GSE101586, GSE101684, and GSE112214 microarrays.

In order to make the study more rigorous, we screened DECs of GSE101586, GSE101684, and GSE112214 microarrays, since there is no intersection between GSE101586 dataset and the other two datasets, we took the intersection of GSE101684 and GSE112214 datasets to obtain a total of eight DECs (Figure 3). The eight overlapped circRNAs were all down-regulated and hsa_circ_0008234 is the most significantly down-regulated.

### 3.2. Interaction of circRNA-miRNA-Target Genes

**Figure 4:**
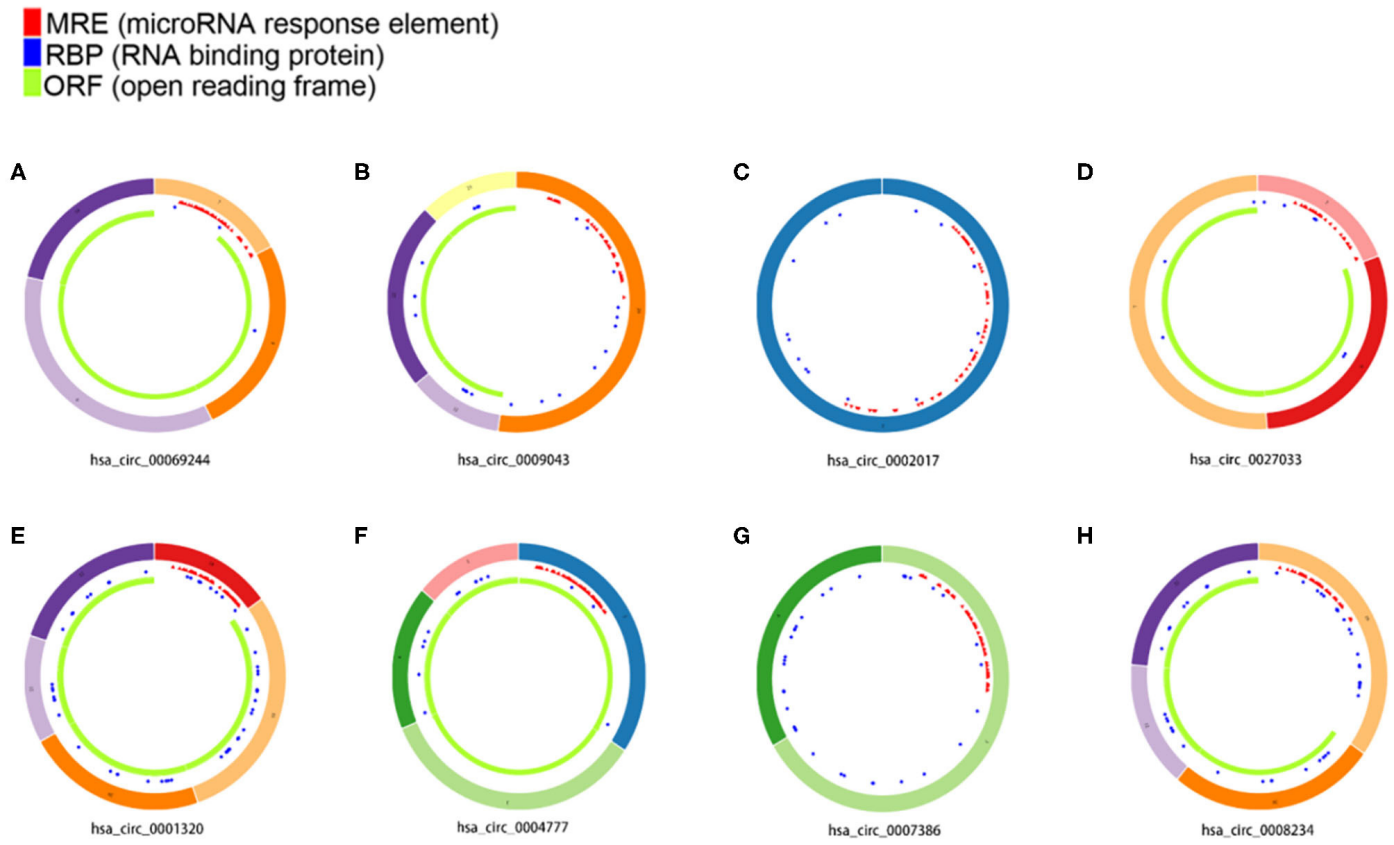
Structural patterns of the eight circRNAs: **(A)** hsa_circ_00069244, **(B)** hsa_circ_0009043, **(C)** _circ_0002017, **(D)** hsa_circ_0027033, **(E)** hsa_circ_0001320, **(F)** hsa_circ_0004777, **(G)** hsa_circ_0007386, **(H)** hsa_circ_0008234.

Bioinformatics analyses show that in mammalian cells, some circRNAs play an important role in miRNA sponges. In this study, the CircBase database gives the location of the gene and chromosome where the circRNA is located, which samples can find the circRNA and fasta base sequences. The basic characteristics of the eight circRNAs are listed in Table 1. The structural patterns of the eight circRNAs are shown in Figure 4. The fluorescent green part represents the protein that circRNA may encode, namely Open Reading Frame (ORF). The blue part represents the approximate location of circRNA binding to the protein and the position of the red small triangle represents the binding position of the circRNA to the miRNA.

**Table 1:** Basic characteristics of the eight differentially expressed circRNAs.

**CircRNA ID**	**Position**	**Genomic length**	**Strand**	**Gene symbol**	**Fold change**
hsa_circ_0009043	Chr2:72945231-72960247	15,016	−	EXOC6B	−1.01962
hsa_circ_0027033	Chr12:56962758-56965639	2,881	+	RBMS2	−1.02682
hsa_circ_0004777	Chr1:204082042-204083733	1,691	+	SOX13	−1.08336
hsa_circ_0002017	Chr2:36623756-36623930	174	+	CRIM1	−1.09055
hsa_circ_0069244	Chr4:16587544-16760883	173,339	−	LDB2	−1.13355
hsa_circ_0007386	Chr2:36668400-36669878	1,478	+	CRIM1	−1.19527
hsa_circ_0001320	Chr3:71064699-71102924	38,225	−	FOXP1	−1.28156
hsa_circ_0008234	Chr3:71090478-71102924	12,446	−	FOXP1	−1.33598

In this study, we chose hsa_circ_0008234, which was down-regulated significantly among eight overlapping DECs, for subsequent target gene prediction and bioinformatics analysis. A total of 43 miRNAs binding to hsa_circ_0008234 were identified.

Based on the miRDB, miRTarBase, and TargetScan databases, the miRNA-target genes contained in the three databases were selected by the miRNA name. This prepares for the next bioinformatics analysis. Totally, 427 target genes were binded to 43 miRNAs, respectively. Table 2 gives the target genes of the first 10 miRNAs in hsa_circ_0008234.

**Table 2:** Target genes of the first 10 miRNAs in hsa_circ_0008234.

**miRNA**	**Target-Genes**
hsa-miR-1272	RTN4,PHKA1,BCAS2,COIL,G2E3,FAM83C,TMEM167A,SMNDC1,SAR1B
hsa-miR-1322	IRF2BPL,C11orf58,CTCF,UCHL5
hsa-miR-150-5p	PDCD4,MTMR9,PDIA6,HILPDA,GK5,ELK1,XPNPEP3,AHI1,EREG,ZEB1,AIFM2
hsa-miR-1910-5p	CLIC4,GSK3B,MTHFD2
hsa-miR-2116-3p	MBD6,EREG,ZBTB18,AP4S1,SRRM4,TXNIP,KRBOX4
hsa-miR-296-3p	TCERG1,HILPDA,ZNF138,ZNF117,GRINA,ZNF99
hsa-miR-31-3p	EPB41L4B,SLC30A5,FRS2,PLEKHB2,CRK,CHMP4B,BACH1
hsa-miR-3677-5p	TBC1D12,SLC7A1,NCMAP,HMGXB3
hsa-miR-4264	ESR1,KLHL15,PALM2,TM4SF1,ADIPOR2,SLCO3A1,SENP1,THRB,FOXK2,PODXL
hsa-miR-4520-3p	PRKX,RAB25,H2AFZ,RAB10,MYOCD,ABAT,NUFIP2

### 3.3. GO and KEGG Pathway Analyses

**Figure 5:**
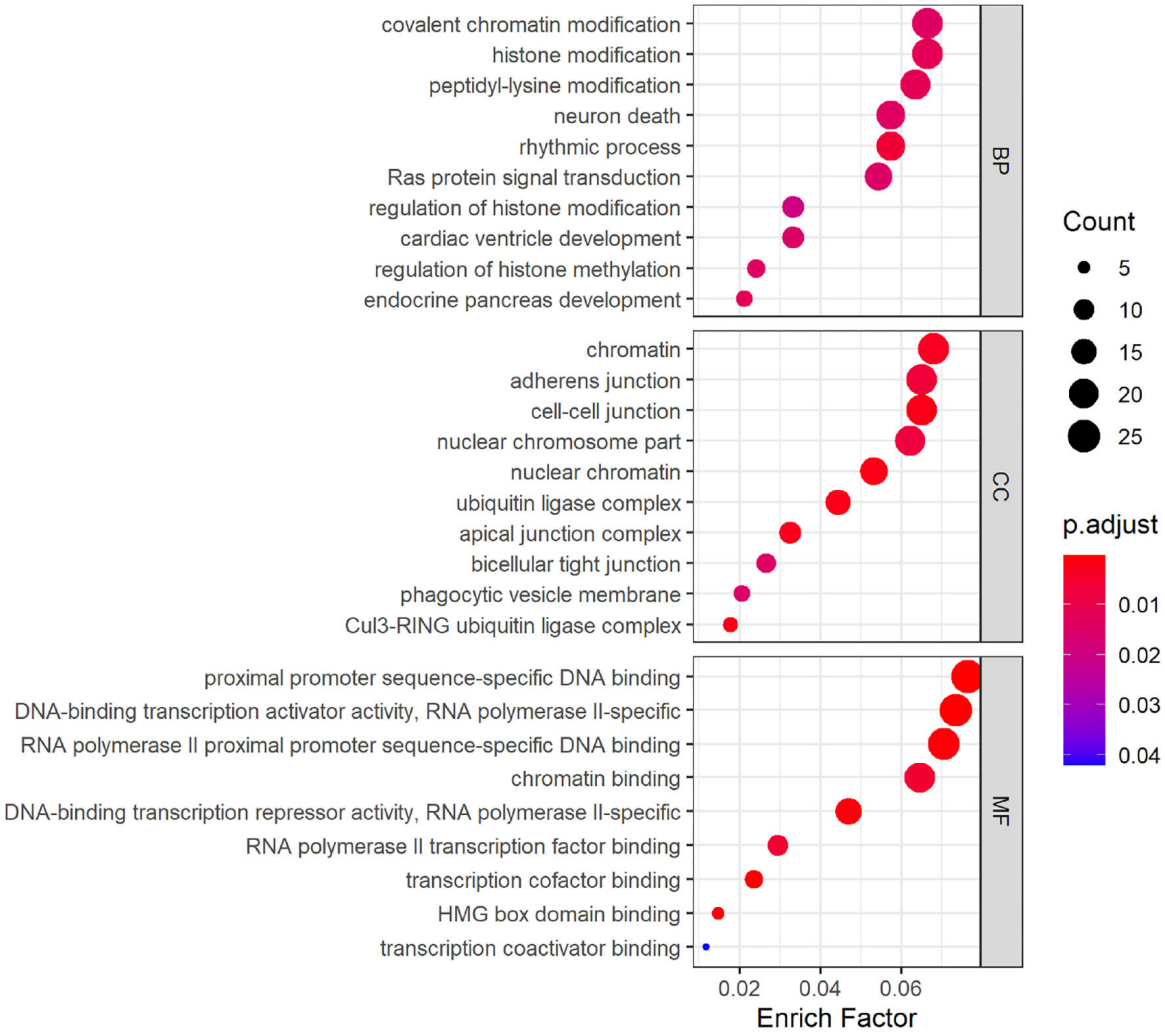
Gene ontology (GO) analysis dotplot of hsa_circ_0008234.

GO analysis was performed on hsa_circ_0008234 (Figure 5), which was down-regulated significantly in overlapping circRNAs. The results showed that the target genes were mainly involved in the biological processes such as “covalent chromatin modification,” “histone modification,” and “peptidyl-lysine modification” etc. As for molecular function, these genes showed enrichment in “chromatin,” “adherens junction,” and “cell-cell junction” etc. Besides, cell component indicated enrichment predominantly at “proximal promoter sequence-specific DNA binding,” “DNA-binding transcription activator activity, RNA polymerase II-specific,” and “RNA polymerase II proximal promoter sequence-specific DNA binding” etc.

**Figure 6:**
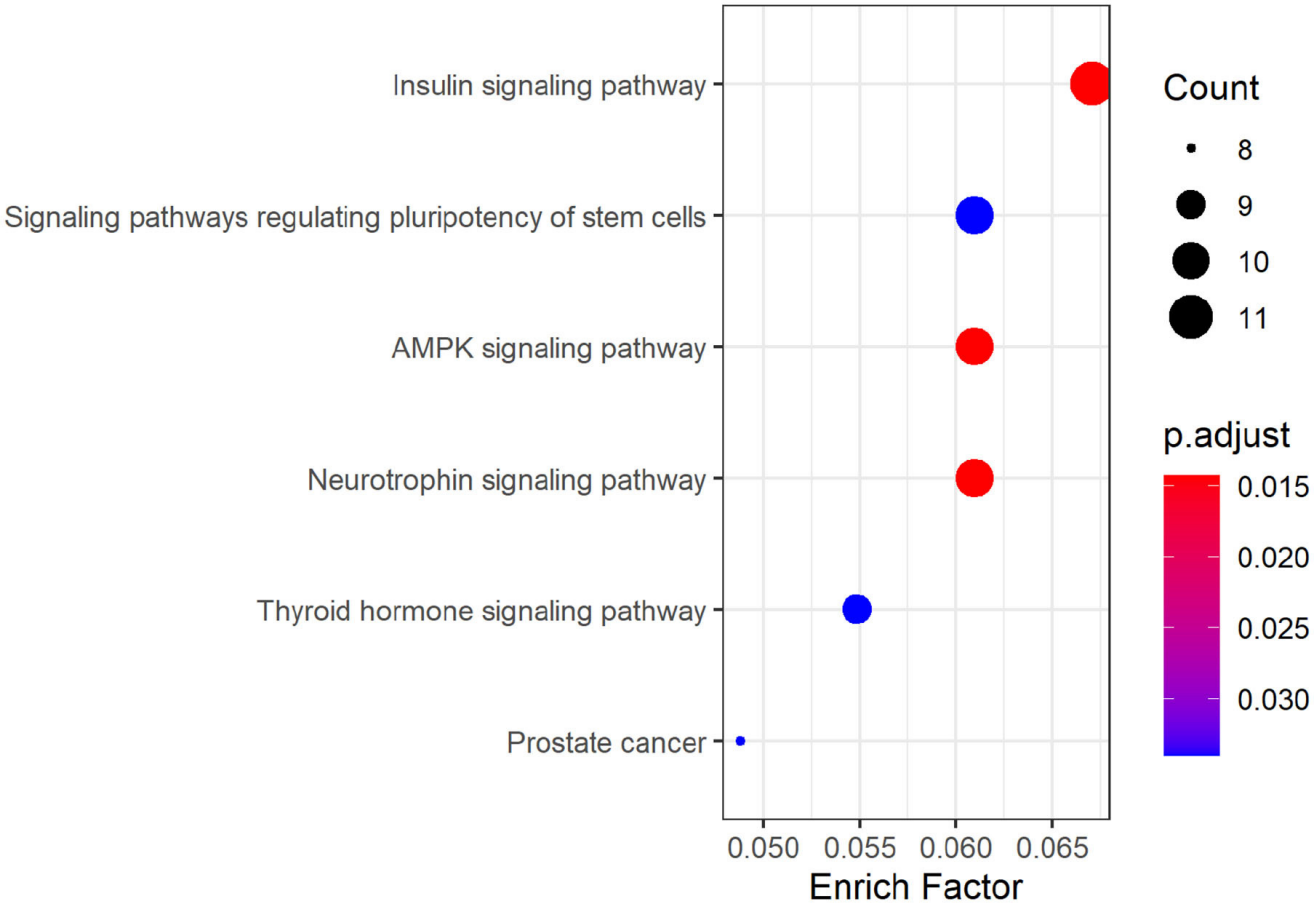
KEGG pathway analysis dotplot of hsa_circ_0008234.

KEGG pathway analysis of hsa_circ_0008234 showed that six significant enrichment pathways include “Insulin signaling pathway,” “Signaling pathways regulating pluripotency of stem cells,” “AMPK signaling pathway,” “Neurotrophic signaling pathway,” “Thyroid hormone signaling pathway,” and “Prostate cancer” (Figure 6).

### 3.4. PPI Network Construction, Module Analysis of Selected Hub Genes

**Figure 7:**
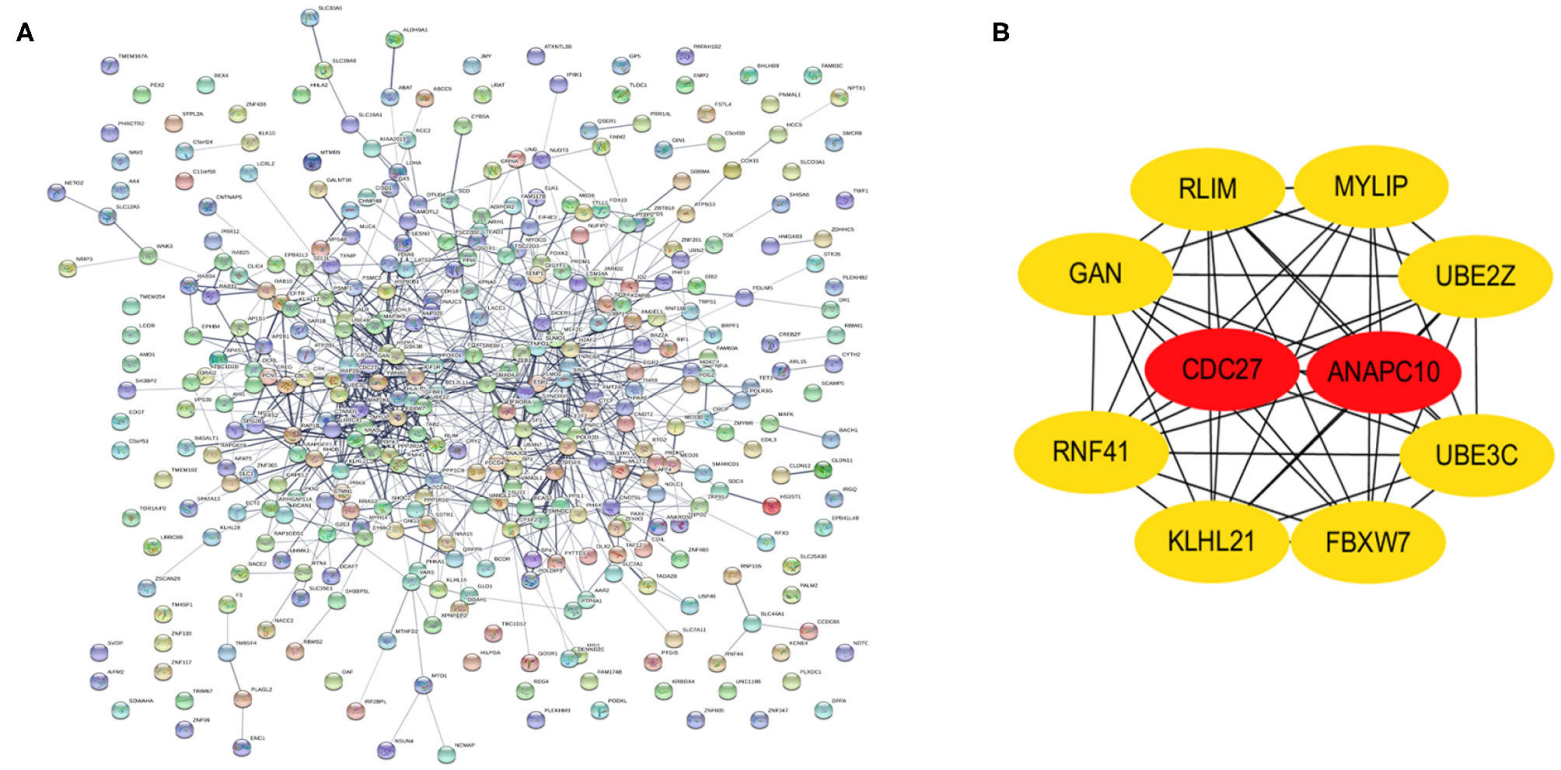
Identification of hub genes from the PPI network with the MCODE and Cytohubba algorithm. **(A)** PPI network of 370 genes. **(B)** PPI network of ten hub genes that extracted from the PPI network.

PPI networks were constructed on the basis of STRING database. For 427 differentially expressed target genes, PPI network was mapped in Figure 7A. In total, 370 nodes and 804 edges were displayed in the PPI network. With the *k*−*core* = 2 as the screening condition, the top 10 hub genes were selected according to the MCC algorithm in Cytohubba. They were RLIM, MYLIP, UBE2Z, UBE3C, FBXW7, KLHL21, RNF41, GAN, CDC27, and ANAPC10 (Figure 7B).

### 3.5. Survival Analysis of Hub Genes

**Figure 8:**
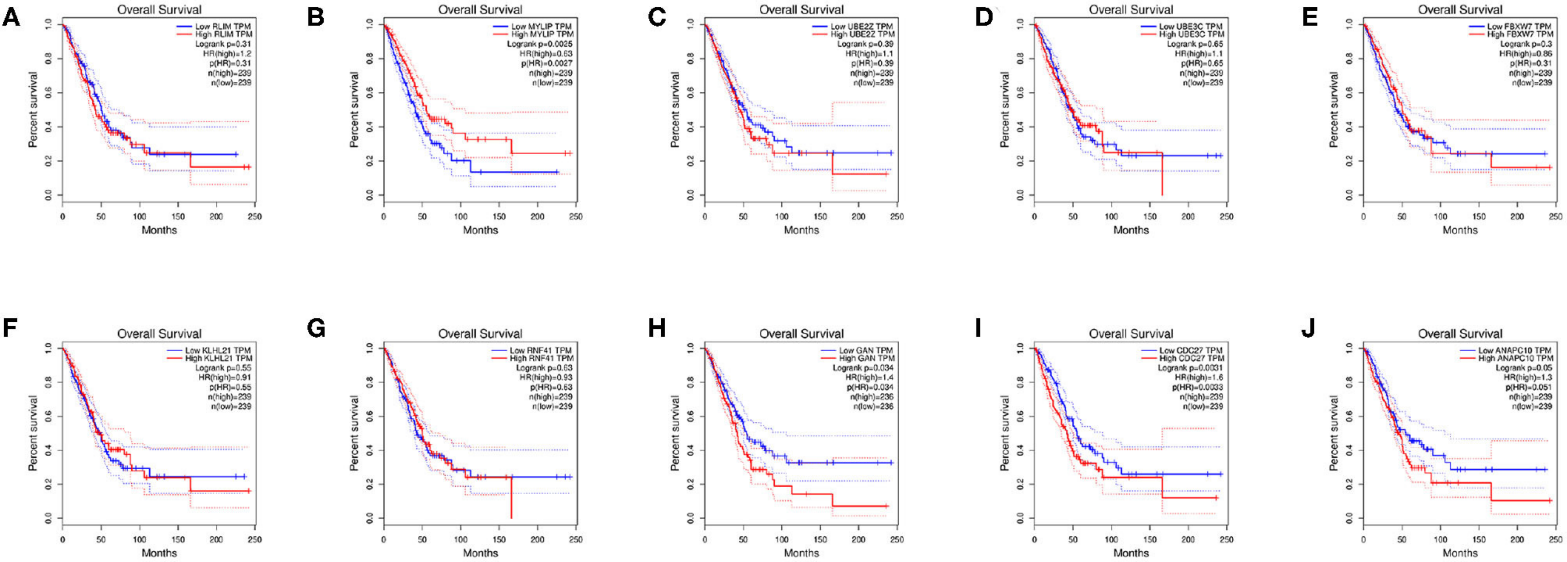
Survival analysis of hub genes, **(A)** RLIM, **(B)** MYLIP, **(C)** UBE2Z, **(D)** UBE3C, **(E)** FBXW7, **(F)** KLHL21, **(G)** RNF41, **(H)** GAN, **(I)** CDC27, **(J)** ANAPC10.

Survival analysis of the hub genes is shown in Figures 8A–J. As shown in the figure below, only MYLIP, CDC27 and GAN among the ten hub genes were found to be significantly associated with patient prognosis (*P* < 0.05) so were chosen as candidate genes.

## 4. Discussion

NSCLC is a type of lung malignant tumor that originates from the bronchial mucosa or glands. NSCLC has a short course and poor prognosis. It is generally found at an advanced stage, which is very difficult to control and treat. CircRNA is a special non-coding RNA with a closed loop structure that abundantly expressed in eukaryotic cells, they are usually expressed in tissues or at developmental stages and most sequences are highly conserved (Wolf et al., 2017). Recently, with the development of gene sequencing data and bioinformatics technology, circRNA has been reported in various types of cancer for diagnosis and treatment. In lung cancer, Cai et al. (2020) analyzed circRNA and gene expression data to detect differentially expressed circRNAs in NSCLC patients, their results explore the pathogenesis of NSCLC to identify novel treatment biomarkers. Liu et al. (2019) identified potential plasma circRNA biomarkers for the diagnosis of lung adenocarcinoma (LUAD), and constructed a ceRNA network to predict the possible mechanisms and function of circRNAs in LUAD. However, the above two studies only used single chip and the samples were less, these results may not be ideally validated in large sample data. Due to the rapid development of gene sequencing technology, a large amount of biological data has been accumulated and some studies have used multiple microarrays to analyse. For example, Li et al. (2020) used the same public datasets as this study, they took the union of GSE101586, GSE101684, and GSE11214 to identify differentially expressed circRNAs among NSCLC and healthy individuals for early diagnosis of NSCLC. Liang et al. (2020) used the intersection of GSE101586, GSE101684 microarrays and GSE104854 high-throughput sequencing data to exploring the aberrant circRNAs and their potential molecular function in LUAD. Although the two GEO microarrays were same as ours, since high-throughput sequencing data and microarray data are different types, the results obtained by overlapping were different. Meanwhile, previous researches mainly focused on the role of circRNA in the early diagnosis of NSCLC, little is known about the expression of miRNA-target genes that combined with circRNAs in the treatment and prognosis of advanced stage NSCLC patients.

In the current study, we used three microarrays to identify 1 DEC in GSE101586, 249 DECs in GSE101684, and 101 DECs in GSE112214. The 8 DECs were screened out from the three sets of microarrays, including hsa_circ_0009043, hsa_circ_0027033, hsa_circ_0004777, hsa_circ_0002017, hsa_circ_0069244, hsa_circ_0007386, hsa_circ_0001320, and hsa_circ_0008234. Based on CircBase database and the CSCD database, 43 miRNAs that bind to hsa_circ_0008234 (the most significant downregulation among 8 circRNAs) were predicted. Moreover, hsa_circ_0008234, was identified previously by Wang et al., who analyzed 40 gallbladder cancer (GBC) patient tissues and normal tissues, suggesting that hsa_circ_0008234 has pleiotropic effects, including promotion of cell proliferation, migration, invasion and inhibition of cell apoptosis in GBC (Wang S. et al., 2019). Additionally, hsa_circ_0008234 acted as the sponge of miR-370 to regulate PKLR, resulting in promoting Warburg effect in GBC progression. The prediction of target genes and enrichment analysis were implemented by multiple bioinformatics tools. Enrichment of these genes affected by DECs, which may provide novel insights for unraveling pathogenesis of NSCLC.

As was suggested by GO analysis, target genes of hsa_circ_0008234 combined with miRNAs in NSCLC were enriched in biological processes of “covalent chromatin modification,” “histone modification,” “neuron death,” “Ras protein signal transduction,” “cardiac ventricle development,” and “endocrine pancreas development” et al. Molecular function of GO analysis showed enrichment “inubiquitin ligase complex,” “nuclear chromatin,” “adherens junction,” “bicellular tight junction,” and “phagocytic vesicle membrane” et al. It is reasonable because frequent cell proliferation and loss of cell adhesion is apparent hallmark of cancers including NSCLC (Hanahan and Weinberg, 2011). In addition, covalent chromatin modification, histone modification and peptidyl lysine modification are the three main epigenetic regulation processes of tumors including lung cancer on the basis of chromatin. These regulatory processes are regulated by a variety of proteins with enzymatic activity and mutations or abnormal expression of these proteins are prone to cause various diseases such as tumors (Liu et al., 2015). KEGG pathway enrichment analysis suggested significant enrichment in pathways including “Insulin signaling pathway,” “AMPK signaling pathway,” and “Neurotrophin signaling pathway” etc. It was consistent with the fact that AMPK can activate the corresponding pathway or inhibit the corresponding pathway and can significantly antagonize NSCLC (Xu et al., 2018).

PPI network of target genes illustrated the overview of their functional connections, of which top10 hub genes were selected: RLIM, MYLIP, UBE2Z, UBE3C, FBXW7, KLHL21, RNF41, GAN, CDC27, ANAPC10. Here, we observed that the survival analysis of MYLIP, CDC27, and GAN reveled a correlation between high expression and poor prognosis. MYLIP is a myosin-regulated light chain interacting protein, which belongs to the cytoskeletal protein cluster and participates in the regulation of cell movement and migration (Zhao et al., 2017). The abbrent expression of MYLIP is seen in the occurrence and development of several diseases. Zhao et al. (2017) found that the regulatory mechanisms of miR-19b promoting breast cancer metastasis through directly targeting MYLIP expression and affecting the expression levels of cell adhesion molecules. Here, we observed that survival analysis of MYLIP revealed a correlation between high expression and poor prognosis. Dysregulation of cell cycle progression plays a key role in tumorigenesis. CDC27 is a core subunit that promotes late complexes/loops. Studies have shown that overexpression of CDC27 promotes the proliferation of colorectal cancer and the cell-dependent approach promotes the ability to transfer and form balls (Qiu et al., 2017). Additionally, Xin et al. (2018) demonstrated that the CDC27 expression was obviously increased in gastric cancer tissues, and significantly correlates with EMT-related biomarkers and poor 5-year overall survival. We speculate that CDC27 participates in the development of NSCLC by regulating the cell cycle, apoptosis, and inflammation levels. Gigaxonin (GAN), an E3 ligase adaptor protein. Given the role of GAN in the ubiquitin proteasome complex, it is possible that it could play a role in oncogenesis. Veena et al. found that GAN directly interacts with NF-κB, and this interaction is dependent on the interaction between p16. It is well-known that p16 is an important tumor suppressor gene that renders cancer cells susceptible to cisplatin treatment. They believe that GAN expression could be the biomarker of cancer (Veena et al., 2014). In our study, survival analysis of GAN found that high expression was associated with poor prognosis. The related regulatory mechanism deserves further exploration.

Bioinformatics analysis of this study suggest that differentially expression circRNAs in NSCLC support the important role of these molecules in the development of NSCLC. Clarifying the underlying mechanisms of the initiation and development of NSCLC would greatly benefit the diagnosis, treatment, and prognosis evaluation. The data in this study provide a new perspective on the mechanisms of lung cancer metastasis and recurrence and highlight potential therapeutic targets that can help improve diagnosis and treatment. Due to the restriction of data and time, this study did not analyze clinical parameters and prognosis. We need further clinical molecular experiments to better confirm the findings of identified genes and pathways in NSCLC that we studied.

## 5. Conclusion

Our study screened differentially expressed circRNAs, miRNAs, and target genes from publicly available microarray data of NSCLC by joint bioinformatics analysis, which can broaden the perspective of the gene study of lung cancer and lay a foundation for further studies on the role of circRNAs in NSCLC. Compared with individual investigation, this study is possible to come up with more reliable and accurate screening results via overlapping relevant data sets. Despite the rigorous bioinformatics analysis of this study, there are still some shortcomings. Further clinical experiments and different gene chip of other platforms are needed to confirm the findings of the identified candidate genes in NSCLC. In conclusion, hub genes including MYLIP, GAN, and CDC27 might serve as biomarkers for precise diagnosis and treatment of NSCLC in the future.

## Data Availability Statement

Publicly available datasets were analyzed in this study. This data can be found at: https://www.ncbi.nlm.nih.gov/gds/?term=GSE101684; https://www.ncbi.nlm.nih.gov/gds/?term=GSE112214; https://www.ncbi.nlm.nih.gov/geo/query/acc.cgi?acc=GSE101586.

## Author Contributions

QS and XL carried out data analysis. MX, LZ, HZ, and YX participated in study design and data collection. PG and LZ conceived the study. All authors read and approved the final manuscript. All authors contributed to the article and approved the submitted version.

## Conflict of Interest

The authors declare that the research was conducted in the absence of any commercial or financial relationships that could be construed as a potential conflict of interest.
